# Associations of Cortical and Subcortical White Matter Morphometric Abnormalities With Clinical and Genetic Findings in *STXBP1* Encephalopathy

**DOI:** 10.1212/NXG.0000000000200416

**Published:** 2026-08-04

**Authors:** Matteo Lenge, Alice Dainelli, Simona Balestrini, Simona Fiori, Davide Mei, Valerio Conti, Letizia Macconi, Lucy Helena Coulter, Amy L. Schneider, Antonio Napolitano, Daniela Longo, Maria Camilla Rossi-Espagnet, Jacopo Proietti, Guja Astrea, Marina Trivisano, Ludovico D'Incerti, Anna Maria Buccoliero, Francesca Darra, Bernardo Dalla Bernardina, Nicola Specchio, Ingrid E. Scheffer, Renzo Guerrini

**Affiliations:** 1 Neuroscience and Human Genetics Department, Meyer Children's Hospital IRCCS, Florence, Italy;; 2 University of Florence, Italy;; 3 Epilepsy Research Centre, Department of Medicine, University of Melbourne, Austin Health, Heidelberg, Victoria, Australia;; 4 Medical Physics Department, Bambino Gesù Children's Hospital, IRCCS, Rome, Italy;; 5 Diagnostic and Interventional Neuroradiology Unit, Bambino Gesù Children's Hospital, IRCCS, Rome, Italy;; 6 Child Neuropsychiatry Unit, Department of Engineering for Innovation Medicine, University of Verona, Italy;; 7 IRCCS Stella Maris Foundation, Pisa, Italy;; 8 Neurology, Epilepsy and Movement Disorders Unit, Bambino Gesù Children's Hospital, IRCCS, Full Member of European Reference Network EpiCARE, Rome, Italy;; 9 Neuroscience and Human Genetics Department, Meyer Children's Hospital IRCCS, Florence, Italy;; 10 Child Neuropsychiatry Unit, AOUI of Verona, Department of Engineering for Innovation Medicine, University of Verona, Full Member of European Reference Network EpiCARE, Verona, Italy;; 11 Research Center for Pediatric Epilepsies (CREP), AOUI of Verona, Italy;; 12 Department of Paediatrics, University of Melbourne, Royal Children's Hospital, Melbourne, Australia; and; 13 Florey Institute and Murdoch Children's Research Institute, Melbourne, Australia.

## Abstract

**Background and Objectives:**

Disease-causing variants in the syntaxin-binding protein 1 (*STXBP1*) gene are among the most common genetic causes of developmental and epileptic encephalopathies and are associated with a wide phenotypic spectrum. Qualitative neuroimaging studies are usually unrevealing or uncover variable MRI findings, including cortical atrophy, thin/dysmorphic corpus callosum (CC), hypo/delayed myelination, and focal cortical dysplasia (FCD).

**Methods:**

We used quantitative MRI methods to estimate abnormal brain properties of the cortical mantle and volume of subcortical structures in patients with *STXBP1* encephalopathy and age- and sex-matched controls.

We performed a region-of-interest group statistical analysis between patients with *STXBP1* encephalopathy and controls by multivariable linear regression models to identify morphometric patterns and to evaluate the effect of the group (patients/controls) on morphometric features. We conducted a longitudinal analysis to estimate the volumetric changes in 4 patients with serial MRI scans at different ages. We calculated the association between the structural alterations and the known *STXBP1* expression levels and explored associations between morphometric and volumetric features and clinical findings (age at seizure onset, intellectual disability [ID]) and genetic variants.

**Results:**

Our analysis included 24 patients and 48 controls and revealed widespread cortical thickening, reduced frontal and occipital surface area, and reduced white matter (left/right hemisphere *p*_value_ = 0.005/0.022) and CC (*p*_value_ < 0.050) volumes. The longitudinal analysis highlighted that brain growth trends were lower than the average trend in the control cohort. In patients with more severe ID, we observed a significantly increased volume of the lateral ventricles (left/right *p*_value_ = 0.049/0.030) and CSF (*p*_value_ = 0.019). Patients with missense variants exhibited more altered morphometric values and more severe reductions of white-matter volumes, possibly because of a dominant negative effect of variants. Two patients were operated for intractable focal seizures, and the histopathologic substrate was FCD-I.

**Discussion:**

The altered cortical patterns and WM reductions we observed in *STXBP1* encephalopathy might be the structural counterpart of the widespread impaired neurotransmitter release caused by a dysfunctional syntaxin-binding protein. The 2 histopathologic observations we describe, bring to 4 the number of reported patients with *STXBP1* encephalopathy and FCD-I, suggesting cortical dyslamination as the architectural substrate for the abnormal morphometric parameters and reduced surface areas.

## Introduction

Syntaxin-binding protein 1 (STXBP1, also known as mammalian uncoordinated-18, Munc18) is a presynaptic protein widely expressed in the brain, particularly during development, and is essential for synaptic vesicle docking and fusion via regulation of the neuronal SNARE complex.^1^

*STXBP1-*knockout mice lack neurotransmitter secretion and face widespread apoptotic neurodegeneration after an apparently normal brain development, suggesting that functional STXBP1 is vital for synapses maintenance, neuronal survival, and neurotransmitter secretion.^2^ Impaired neurite growth has been observed both in animal models^3^ and human induced pluripotent stem cell (iPSC)-derived neurons from heterozygous patients with *STXBP1* developmental and epileptic encephalopathy (DEE).^4^

Pathogenic heterozygous *STXBP1* gene variants are among the most common genetic causes of DEEs.^5,6^ While most pathogenic variants are de novo heterozygous, rare patients with biallelic variants have also been described.^7^ The phenotypic spectrum of *STXBP1* encephalopathy is wide,^8^ encompassing almost invariably developmental delay, often within an early infantile developmental and epileptic encephalopathy (EIDEE), DEE (DEE4, OMIM # 612164), and intellectual disability (ID) without epilepsy.^9^ Altered motor function (hypotonia, spasticity) and movement disorders (mainly tremor, stereotypies, ataxia, dyskinesia) are common accompanying comorbidities.^10,11^ Within the spectrum of *STXBP1* encephalopathy, neither strong clear-cut genotype-phenotype correlations nor overarching phenotypic similarities have been reported, despite numerous recurrent variants.^8^

Standard clinical brain MRI has been described as normal in about 50%–70% of patients with *STXBP1* encephalopathy; in the remaining patients, reported MRI findings include cortical atrophy, thin or dysmorphic corpus callosum (CC), hypo/delayed myelination,^11-13^ and focal cortical dysplasia (FCD).^13-15^ In 2 reported patients, epilepsy surgery with resection of MRI-visible FCD, demonstrated either FCD Ia^14^ or Ib^15^. These observations testify abnormal cortical lamination in the samples studied but provide limited insight into the overall brain structure because co-occurrence of an area of epileptogenic dysplasia in *STXBP1* encephalopathy might still be related to chance and the nosology of FCD-I is debated because of lack of clear clinical and pathologic correlations and variable underlying etiologies.^16^

Quantitative neuroimaging techniques able to detect overall or even discrete structural brain alterations have not been applied to patients with *STXBP1* encephalopathy. For instance, in another form of epileptogenic developmental brain disorder, *PCDH19* encephalopathy, we showed morphometric patterns of structural alterations to correlate with clinical manifestations and gene expression patterns.^17^ Likewise, we applied quantitative MRI in patients with *SCN1A-*related epilepsies and found bilateral atrophic changes in the temporo-limbic cortex, amygdala, and hippocampus, with Dravet syndrome being associated with more severe atrophy patterns compared with the milder genetic epilepsy with febrile seizures plus (GEFS+) phenotype.^18^

We hypothesized that morphometric brain abnormalities, not detectable through standard neuroimaging, might contribute to phenotype severity in *STXBP1* encephalopathy. To investigate this hypothesis, we used morphometric methods to identify abnormal brain MRI patterns and performed a longitudinal analysis in a small subset of patients, and a comparative analysis of patient subgroups stratified by varying degrees of clinical severity and different genetic variants.

## Methods

### Standard Protocol Approvals, Registrations, and Patient Consents

The study was conducted in accordance with the Declaration of Helsinki and was approved by the Pediatric Ethics Committee of the Tuscany Region (MRI4-DEE, no. CET_76/2023), in the context of the DESIRE project and its extension by the DECODE-EE and the Human Brain Optical Mapping projects (details are provided in the “Funding” section) and the experiments were undertaken with the understanding and written consent of each subject or their guardian. We obtained written informed consent to access and use pseudonymized clinical, neuroimaging and genetic data, from all participants' parents or legal guardians.

### Participants

We enrolled eligible individuals with *STXBP1* encephalopathy and age- and sex-matched controls followed at 5 tertiary pediatric neurology centers in Italy and Australia. Inclusion criteria for patients enrollment were (1) pathogenic or likely pathogenic variant in the *STXBP1* gene (canonical transcript NM_003165.6), identified by next-generation sequencing, confirmed through Sanger sequencing, and classified according to the American College of Medical Genetics and Genomics criteria^19^; (2) at least one 3T brain MRI examination suitable for morphometric analysis (3D T1-weighted isotropic MRI); and (3) age between 1 and 18 years at the time of brain MRI scan.

We collected clinical, genetic, neuroimaging, and electrophysiologic data using a standardized template shared by all 5 recruiting centers (Table 1 and eTable 1). Information included details on patients' epilepsy phenotype, psychiatric comorbidities, cognitive profile, neuropsychological testing, and expert review of genetic findings and structural MRI scans. We classified ID at last follow-up as mild, moderate, severe, or profound, according to either clinical or formal cognitive assessments, or both^20^ (details in eMethods). For patients who proceeded to epilepsy surgery, we classified brain tissue histopathology according to the 2022 International League Against Epilepsy consensus classification method for FCD.^16^

**Table 1:** Genetic and Clinical Data of Patients With *STXBP1* Variants

No.	Age at last follow-up (y)/Sex	*STXBP1* (*NM_003165.6*) *variant*	Age at seizure onset (mo)	Age at MRI (y)	ASMs at MRI	Intellectual disability (grade)	Drug-resistant epilepsy/Engel score
1	13/F	c.1622_1627del p.Ile541_Gly543delinsArg	No seizure	3.7	0	Moderate	No epilepsy
2	14/M	c.706G>C p.Gly236Arg	13	6.8	VPA, CLB	Mild	Yes
3	18/M	c.1012C>T p.Gln338*	2	4	0	Severe	Yes
4	20/M	c.1A>T p.?	132	12.2	CBZ	Moderate	No
5	12/F	c.1565G>A p.Trp522*	4	2.3	NZP, LEV, VGB	Severe	No
6	12/F	c.57_59del p.Ile19_Lys20delinsMet	11	3.4	LEV	Mild	Yes/1
7	14/F	c.1099C>T p.Arg367*	1	4.1	VPA	Profound	No
8	9/M	c.56T>C p.Ile19Thr	19	4.4	CBZ, CLB, LTG	Moderate	Yes/4
9	15/M	c.419_420dupAT p.Glu141Metfs*25	0.16	9.1	VPA, CLB	Severe	No
10	26/M	c.764A>G p.Asp255Gly	14	12.5	CLN, TPM	Moderate	Yes
11	33/M	c.1217G>A p.Arg406His	0	9.1	LCS, VPA	Severe	Yes
12	5/F	c.814delG p.Glu273fs*3	2	4.1	LEV, VPA	Severe	Yes
13	8/M	c.1250-2A>G splicing	2	6.7	LEV, ACTH	Moderate	No
14	16/F	c.1262delA p.Asn422Thrfs*2	0.16	15.2	CLB, LEV, VGB	Severe	Yes
15	7/M	c. 440T>G p.Leu147Trp	0.03	5.8	VGB, ACTH	Severe	Yes
16	19/M	c.1324A>G p.Asn442Asp	24	18	CBZ	Severe	No
17	9/M	c.993_995delGAA p.Lys332del	1	7.9	LEV	Severe	No
18	14/F	c.429+5G>A	NA	12.4	VPA	Moderate	No
19	21/M	c.1216C>T p.Arg406Cys	5.3	5.3	VPA	Severe	No
20	6/F	c.1006C>T p.Gln336*	NA	3.2	CBZ	Moderate	No
21	20/M	c.897_898del p.Ser300ProfsTer13	1.5	6	CBZ, VPA, CLB	Profound	Yes
22	10/M	c.1708A>G p.Thr570Ala	6	2	TPM, LEV, CLB	Severe	Yes
23	4/F	9q33.3–34.11 (chr9:129079208-130851795) deletion	2.5	1.5	VGB, TPM	Severe	No
24	15/M	c.323_325+1del	19	7.6	LEV, VGB	Severe	Yes

Abbreviations: ACTH = adrenocorticotropic hormone; ASMs = antiseizure medications; CBZ = carbamazepine; CLB = clobazam; Engel score: 1 = class I, seizure free or no more than a few early, nondisabling seizures, or seizures on drug withdrawal only; 4 = class IV, no worthwhile improvement, some reduction, no reduction, or worsening are possible; F = female; LCS = lacosamide; LEV = levetiracetam; LTG = lamotrigine; M = male; NA = not available; NZP = nitrazepam; TPM = topiramate; VGB = vigabatrin; VPA = valproic acid (valproate).

To perform between-group morphometric analysis, we enrolled a cohort of controls, sex- and age-matched with patients, including healthy individuals who were investigated by MRI for because of either uncomplicated headache or minor head trauma. All controls had normal neurologic examination and documented normal subsequent neurodevelopment.

### MRI Data Acquisition

Participants underwent MRI acquisitions using nonstandardized imaging protocols on 1.5T and 3T MR systems. For structural brain assessment, all scans included at least a 3D T1-weighted fast-spoiled gradient echo (3D-T1), 3D T2-weighted fluid attenuation inversion recovery (3D T2-FLAIR), T2-weighted Fast Spin-Echo, T2*-weighted Gradient-echo, and 2D T1-weighted acquisitions.

### Data Analysis

#### MRI-Based Visual Analysis

We examined diagnostic brain MRI images to identify gross structural abnormalities and artifacts that could potentially interfere with computational analyses.

#### MRI-Based Cortical, Subcortical, and White Matter Brain Analysis

We conducted morphometric analysis of cortical and subcortical gray matter (GM) structures by processing T1-3D MR images of patients and controls using the FreeSurfer pipeline (version 7.2.0). The processing pipeline included the following steps^21^: segmentation of subcortical structures; segmentation of cortical structures and surface reconstruction of boundaries between white and gray matter (*white* surface) and between gray matter and CSF (*pial* surface); parcellation of the cortical surfaces to divide the cortex into different gyral and sulcal regions. We obtained 3D high-resolution reconstructions of *white* and *pial* surfaces for each hemisphere. To ensure accuracy, we conducted visual inspections and manually corrected the subcortical segmentations and cortical surface reconstructions as needed.

We measured the volume of subcortical structures and the intracranial, subcortical, and cortical whole brain volumes.

We estimated the morphometric properties of the cortex by adopting a region of interest (ROI) approach. We assessed the cortical thickness (CT), surface area (SA), and cortical volume (CV) for each vertex of the cortical surfaces. CT is estimated as the distance between *white* and *pial* surface and is related to neural migration/postmigrational organization and gray matter pruning.^22^ SA is defined as the sum of the area of the triangles surrounding a vertex and has been associated with cognitive abilities and demonstrated a high degree of heritability.^23^ CV is obtained by multiplying the values of CT and SA.

We calculated the average values of morphometric features (CT, SA, and CV) for the ROIs of the parcellated cortex.^24^ We also performed white matter segmentation based on the neighboring ROIs of the parceled cortex and assessed the volume of each ROI.

#### Longitudinal MRI-Based Analysis on Single Individuals

We conducted a longitudinal analysis to estimate the percentage volumetric changes in 4 patients with serial MRI scans at different ages.

### Statistical Analysis

We conducted statistical analyses on morphometric features using FreeSurfer^21^ tools and a script developed in MATLAB R2023b (Statistics and Machine Learning Toolbox Version 23.2, MathWorks Inc., Natick, MA).

### Group-Related MRI-Based Cortical and Subcortical Statistical Analysis

We performed a ROI-based statistical analysis to identify group-related patterns in morphometric features (CT, SA, and CV). To evaluate the effect of the diagnostic group (*Group*) and age (*Age*) on morphometric features (*Morph*), we performed a group analysis between patients and controls by a multivariable linear regression model, using the center of acquisition (*Center*) and the age (*Age*) as covariates (Morph = β_0_ + β_1_ Center + β_2_ Group + β_3_ Age + β_4_ Handedness). We adopted the center (*Center*) as a confounding variable to model the variability introduced by the different MRI scanners, and handedness (*Handedness*) to assess confounding effects coming from laterality. We considered 0.05 as the threshold for significant *p*_values_ and corrected for multiple comparisons.

### Comparisons Between Morphometric Analysis vs Gene Expression Patterns

For each ROI, we extracted the spatial STXBP1 expression levels from the brain-wide Allen Human Brain Atlas,^25^ which includes measurements from more than 20,000 genes, obtained through microarray technology and sampled across 3,702 spatially distinct tissue samples. We calculated the spatial correlation between the structural SA and CT alterations (*β*_2_) and the *STXBP1* expression levels and assessed significance testing by spin permutation tests (with 1,000 rotations, significance threshold *p*_spin_ < 0.1).

### Comparisons Between Morphometric Analysis vs Clinical and Genetic Variables in *STXBP1* Encephalopathy

For each patient, we investigated the relationships between morphometric and volumetric features and clinical variables (*age at seizure onset, drug-resistant epilepsy, intellectual disability, autism, corticosteroid treatment*), using the following linear regression model: *Clinics* = *β*_0_ + *β*_1_ *Center* + *β*_2_ *Morph* + *β*_3_ *Age*. We also correlated morphometric features with the type of genetic variant, comparing patients harboring missense variants against a combined group of nonmissense variants, including protein-truncating variants (*PTV*), whole or partial *STXBP1* gene deletions (*del*), splice-site variants (*splicing*), and start loss variants (*start loss*). This specific comparison was chosen building on previous work showing consistent phenotypic similarities within the “nonmissense” group when compared with the other^17,18^ and to test the hypothesis that missense variants might exert a dominant negative effect, potentially leading to more severe phenotypes than the haploinsufficiency typically associated with the other variant types.

### Data Availability

Anonymized data not published within this article will be made available by request from any qualified investigator.

## Results

### Overall Clinical Correlations

We studied 24 patients and 48 controls. Nine patients had been included in previous publications.^11,12,26-30^ We compared age distributions of individuals with *STXBP1* encephalopathy (mean age 6.6 years, median 5.6, range 0.9–18.2) and age- and sex-matched controls (±1 year, mean age 6.3 years, median 4.7, range 1.0–18.8) and found no significant age differences (*p*_value_ = 0.75) at the time of MRI. We found a statistically significant correlation between age at seizure onset and ID (*r*_value_ = −0.66, *p*_value_ = 0.005).^11,12,26-30^ Clinical and genetic information on our patient cohort is summarized in Table 1 and comprehensively reported in eTable 1A; neuroimaging, neurophysiologic, cognitive, and psychiatric information is reported in eTable 1B.

### Visual MRI Analysis

**Figure 1:**
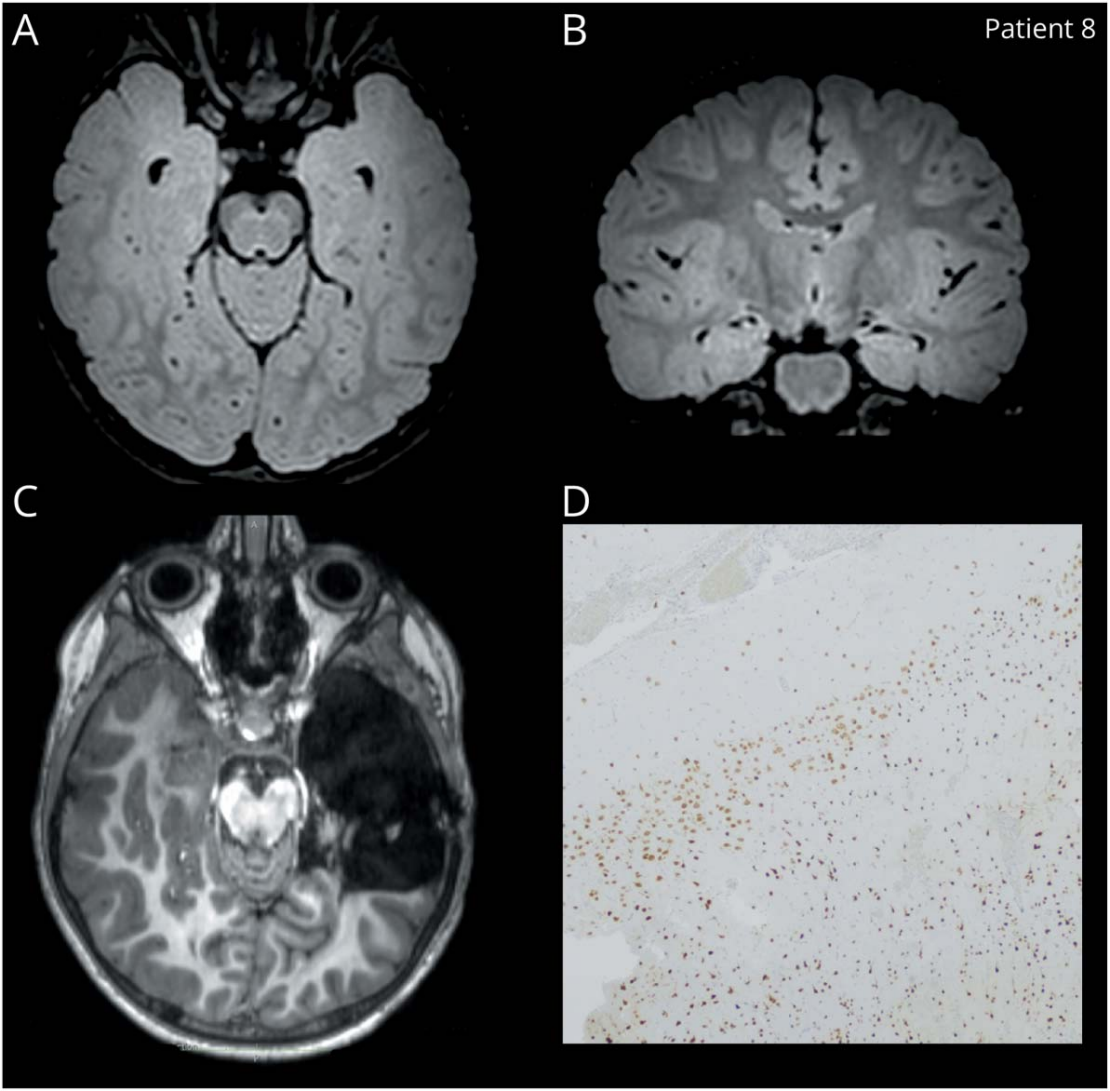
Visual MRI Analysis in a Patient With *STXBP1* Variants (Patient 8) White-gray matter blurring in the left anterior temporal lobe, as revealed by axial (A) and coronal (B) 3D-FLAIR MRI sections. After surgical resection (C, 3D-T1 MRI section), histopathology (D, original magnification ×20) was consistent with type I focal cortical dysplasia. FLAIR = fluid attenuation inversion recovery; *STXBP1 =* syntaxin-binding protein 1.

After visual inspection of MRI sequences, we could complete morphometric analysis for all controls and patients. Nine of 24 (37.5%) patients had visually normal MRI (eTable 2 and Figure 1A). In the remaining patients (15/24, 62.5%), we observed MRI abnormalities (eFigure 1), including CC thinning (7/15, 46.7%), WM reduced signal intensity (8/15, 53.3%), and white/gray matter blurring suggestive of FCD (1/15, 6.7%). In 2 patients (patients 6 and 8) brain MRI suggested left anterior temporal FCD. Following surgical resection of the affected region, histopathologic analysis confirmed FCD-I (Figure 1). The left temporal lobes of these 2 patients were excluded from the ROI morphometric analysis.

### Group-Related MRI-Based Morphometric Analysis of Cortical Structures

**Figure 2:**
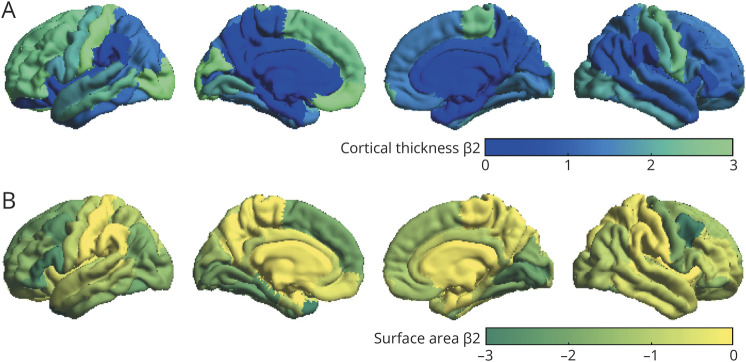
Statistical ROI-Based Group Analysis of Patients With *STXBP1* Encephalopathy Compared With Age- and Sex-Matched Controls The results of the statistical analysis on cortical thickness (CT, panel A) and surface area (SA, panel B) are represented by *β*_*2*_ maps superimposed on the pial surfaces of the left and right hemispheres. We observed statistically significant higher values of CT and lower values of SA in fronto-temporo-occipital regions (for further details, please see Table 2). ROI = region of interest; SA = surface area; *STXBP1 =* syntaxin-binding protein 1.

We performed a surface-based morphometric analysis (eFigure 2) in all 24 patients and 48 controls and found significant and widespread left-hemispheric increase in CT compared with controls (left/right *p*_value_ = 0.052/0.282) (Table 2 and Figure 2).

**Table 2:** Effect of Group on Cortical Thickness, Surface Area, and Cortical Volume of Structures of the Cortex

		Morphometric variables					
		Cortical thickness [mm]		Surface area [mm^2^]		Cortical volume [mm^3^]	
Region	Side	_β2_	*p*_value_	_β2_	*p*_value_	_β2_	*p*_value_
Entorhinal	L	−0.20	0.834	−0.79	0.429	−0.42	0.673
	R	−1.27	0.210	1.18	0.243	0.81	0.420
Fusiform	L	1.52	0.134	−1.47	0.146	0.37	0.716
	R	1.33	0.188	−1.00	0.322	0.72	0.473
Parahippocampal	L	−0.79	0.434	−1.63	0.108	−1.37	0.175
	R	−0.55	0.581	0.10	0.921	0.56	0.577
Superior temporal	L	1.23	0.223	0.16	0.872	1.16	0.250
	R	1.22	0.228	0.94	0.348	1.30	0.199
Middle temporal	L	1.98	0.052	−1.03	0.306	1.35	0.181
	R	0.96	0.343	0.49	0.623	0.82	0.413
Inferior temporal	L	1.25	0.215	−0.84	0.402	0.34	0.734
	R	1.43	0.157	0.35	0.731	2.41^a^	0.019^a^
Temporal pole	L	1.14	0.258	−2.17^a^	0.034^a^	1.14	0.260
	R	−0.36	0.722	−0.52	0.604	−0.53	0.601
Frontal pole	L	2.36^a^	0.021^a^	−1.47	0.145	1.72	0.091
	R	1.02	0.313	−0.94	0.350	2.48^a^	0.016^a^
Medial orbito frontal	L	3.08^a^	0.003^a^	0.02	0.984	2.57^a^	0.012^a^
	R	1.42	0.159	−0.64	0.527	1.83	0.071
Caudal middle frontal	L	2.46^a^	0.017^a^	−1.93	0.057	−0.17	0.867
	R	0.45	0.656	−3.11^a^	0.003^a^	−2.25^a^	0.028^a^
Lateral orbito frontal	L	−0.01	0.992	−0.47	0.640	0.45	0.653
	R	0.34	0.735	−0.50	0.616	0.84	0.402
Rostral middle frontal	L	2.12^a^	0.037^a^	−1.70	0.094	0.04	0.965
	R	1.22	0.228	−0.36	0.723	0.46	0.647
Pars opercularis	L	1.36	0.180	−2.20^a^	0.031^a^	−0.86	0.393
	R	1.24	0.220	−1.46	0.150	0.37	0.714
Pars orbitalis	L	1.85	0.069	−1.96	0.054	0.31	0.754
	R	0.70	0.487	−0.90	0.369	−0.43	0.671
Pars triangularis	L	1.92^a^	0.059^a^	−2.94^a^	0.004^a^	−0.79	0.431
	R	−0.40	0.690	−1.17	0.244	0.02	0.984
Superior frontal	L	2.33^a^	0.022^a^	−1.58	0.120	1.04	0.300
	R	1.07	0.289	−0.75	0.453	1.15	0.254
Precentral	L	1.89	0.063	−0.40	0.690	1.61	0.113
	R	2.30^a^	0.024^a^	−1.09	0.280	1.00	0.322
Postcentral	L	1.98	0.052	0.77	0.442	2.50^a^	0.015^a^
	R	2.06^a^	0.044^a^	0.78	0.439	1.77	0.081
Paracentral	L	0.30	0.768	0.84	0.403	0.83	0.411
	R	2.49^a^	0.016^a^	1.43	0.156	1.93	0.057
Supramarginal	L	−0.28	0.777	1.35	0.181	1.40	0.166
	R	−0.46	0.649	1.59	0.300	1.39	0.170
Superior parietal	L	0.58	0.567	−1.49	0.141	−0.51	0.609
	R	0.54	0.591	−0.49	0.623	0.16	0.876
Inferior parietal	L	0.37	0.714	−2.06^a^	0.043^a^	−1.35	0.180
	R	0.17	0.862	−0.44	0.664	−0.27	0.787
Cuneus	L	2.33^a^	0.023^a^	−1.06	0.291	0.81	0.422
	R	0.44	0.660	−1.61	0.113	−0.51	0.614
Precuneus	L	−0.46	0.646	−0.15	0.882	−0.07	0.948
	R	0.08	0.935	−0.19	0.852	0.40	0.690
Lateral occipital	L	2.87^a^	0.005^a^	−1.06	0.294	1.24	0.220
	R	1.57	0.120	−0.76	0.451	0.57	0.570
Lingual	L	1.71	0.091	−1.55	0.126	0.48	0.633
	R	1.35	0.181	−2.04^a^	0.045^a^	0.17	0.868
Pericalcarine	L	0.34	0.737	−1.97	0.053	−1.06	0.293
	R	1.10	0.277	−2.15^a^	0.035^a^	−0.88	0.383
Insula	L	0.53	0.601	0.12	0.908	0.99	0.326
	R	0.28	0.783	0.56	0.575	1.26	0.214
Whole cortex	L	1.98	0.052	−1.26	0.213	−1.11	0.270
	R	1.08	0.282	−0.67	0.505	−2.61^a^	0.011^a^

Effect of group (individuals with *STXBP1*-related encephalopathy or controls) on cortical thickness, surface area, and cortical volume of ROI cortical structures, located in the left (L) and right (R) hemispheres. Data were analyzed by a multivariate linear regression model (Morph = *β*_0_ + *β*_1_ Center+ *β*_2_ Group+ *β*_3_ Age+ *β*_4_ Handedness) to explore the effect on morphometric values because of the center of acquisition (Center), group (Group), age (Age), and handedness (Handedness).

a Indicates significantly altered measures (*p* < 0.05).

In the frontal regions, we observed reduced SA, including the left caudal middle frontal (*p*_value_ = 0.057), *pars opercularis* (*p*_value_ = 0.031), *pars orbitalis* (*p*_value_ = 0.054), and *pars triangularis* (*p*_value_ = 0.004) gyri, the temporal pole (*p*_value_ = 0.034), and the right lingual (*p*_value_ = 0.045) and pericalcarine (*p*_value_ = 0.035) gyri. SA was also reduced, although not significantly, at the whole-brain level (left/right *β*_2_ = −1.26/-0.67, *p*_value_ = 0.213/0.505).

As regards cortical volumes (eFigure 3A), we observed a whole-brain group-related reduction, with no significant difference between patients and controls.

### Group-Related MRI-Based Volumetric Analysis of Subcortical Structures

We observed volumetric reduction in the subcortical structures of patients compared with controls (Table 3). The pattern of reduction involved WM bilaterally (left/right *p*_value_ = 0.005/0.022, eFigure 3B), CC in its anterior (*p*_value_ < 0.001), middle anterior (*p*_value_ = 0.009) and posterior (*p*_value_ = 0.005) sections, and the hippocampi, although not significantly (left/right *p*_value_ = 0.074/0.425). We also noticed increased volumetric values of the WM hypointensity regions (*p*_value_ = 0.048), lateral ventricles (left/right *p*_value_ = 0.012/0.011) and CSF (*p*_value_ = 0.070). The distributions of estimated intracranial volumes (eICV) were not statistically different between patients and controls (*p*_value_ = 0.409). For group-related patterns of the WM volume (eFigure 3B), the increase was slightly lower in *STXBP1* patients than in controls.

**Table 3:** Effect of Group on Subcortical Volumes

Region	Side	Controls		Patients		*β*_2_	*p*_value_
		Mean	STD	Mean	STD		
Thalamus	L	7,090.03	876.65	6,834.47	973.52	−1.73	0.089
	R	6,892.43	809.62	6,713.92	1,002.3	−1.14	0.258
Caudate	L	3,536.03	472.28	3,502.91	461.43	−0.34	0.735
	R	3,721.5	477.97	3,592.48	452.21	−1.20	0.235
Putamen	L	5,046.38	527.69	4,801.32	864.04	−1.74	0.086
	R	5,116.81	595.65	4,864.62	840.34	−1.81	0.075
Pallidum	L	1771.25	289.63	1808.84	344.78	0.59	0.560
	R	1723.77	258.25	1811.78	367.84	1.40	0.165
Lateral ventricle	L	5,477.3	3,065.18	8,334.82	6,630.92	2.59^a^	0.012^a^
	R	4,591.85	2,416.26	6,677.62	4,581.89	2.62^a^	0.011^a^
Hippocampus	L	3,809.79	402.47	3,678.69	582.08	−1.81	0.074
	R	3,916.53	449.64	3,859.47	537.92	−0.80	0.425
Amygdala	L	1,578.32	240.71	1,560.25	265.25	−0.44	0.660
	R	1,667.09	239.94	1,682.38	325.9	0.15	0.883
CSF		834.88	169.67	1,029.59	384.84	3.08^a^	0.003^a^
Cerebellum WM	L	12,080.6	2,906.04	11,211.22	2,142.18	−1.53	0.130
	R	11,235.1	2061.21	10,630.34	2,280.61	−1.89	0.062
Cerebellum GM	L	52,515.18	5,664.83	52,394.92	6,323.75	−1.64	0.105
	R	55,018.22	5,486.41	52,553.95	6,455.71	−1.92	0.059
Posterior CC		770.88	164.7	667.78	214.35	−2.90^a^	0.005^a^
Middle posterior CC		433.93	94.33	392.21	123.97	−1.95	0.055
Central CC		479.38	103.97	434.37	135.96	−1.88	0.064
Middle anterior CC		460.45	99.69	402.75	126.94	−2.70^a^	0.009^a^
Anterior CC		813.09	119.06	658.19	136.42	−5.84^a^	<0.001^a^
Brain stem		16,876.38	2,821.44	16,304.07	3,250.45	−1.62	0.109
Brain volume	L	276,318.65	31,395.45	279,639.81	34,231.49	0.91	0.363
	R	278,164.85	32,389.46	281,374.16	35,503.92	0.87	0.389
Subcortical GM		55,977.32	5,540.52	54,491.12	7,404.44	−1.59	0.117
Total GM		719,403.74	70,774.64	720,141.64	79,403.2	0.31	0.755
White matter	L	183,028.35	31,234.16	169,986.92	33,914.74	−2.93^a^	0.005^a^
	R	182,672.69	30,984.11	172,731.96	32,791.95	−2.34^a^	0.022^a^
eICV		1,373,963.66	159,016.96	1,375,631.46	209,348.23	0.83	0.409

Abbreviations: CC = corpus callosum; GM = grey matter; WM = white matter.

Effect of group (*Group,* individuals with *STXBP1*-related encephalopathy or controls) on the volume (mm^3^) calculated in the subcortical regions, located in the left (L) and right (R) hemispheres. Data were analyzed by a multivariate linear regression model (*Volume* = *β*_0_ + *β*_1_ *Center* + *β*_2_ *Group* + *β*_3_ Age + *β*_4_ *Handedness* + *β*_5_ *eICV*) to explore the effect on the volume because of the center of acquisition (*Center*), group (*Group*), age (*Age*), handedness (*Handedness*), and estimated intra-cranial volume (*eICV*).

a Indicates significantly altered measures (*p* < 0.05).

### MRI-Based Volumetric Analysis of White Matter ROIs Segmentations

The comparison of volumetric analysis of WM segmentations between patients and controls revealed that WM reductions observed in patients at whole-brain level were much more pronounced in the regions underlying the frontal lobes bilaterally (eTable 2). We estimated statistically significant reductions in the superior frontal (left/right *p*_value_ = 0.011/0.049), medial (left/right *p*_value_ = 0.329/0.006), lateral orbito-frontal (left/right *p*_value_ = 0.009/0.065), caudal (left/right *p*_value_ = 0.088/0.001), rostral middle-frontal (left/right *p*_value_ = 0.009/0.144), *pars opercularis* (left/right *p*_value_ = 0.003/0.006), and right precentral (*p*_value_ = 0.028) WM ROIs.

**Figure 3:**
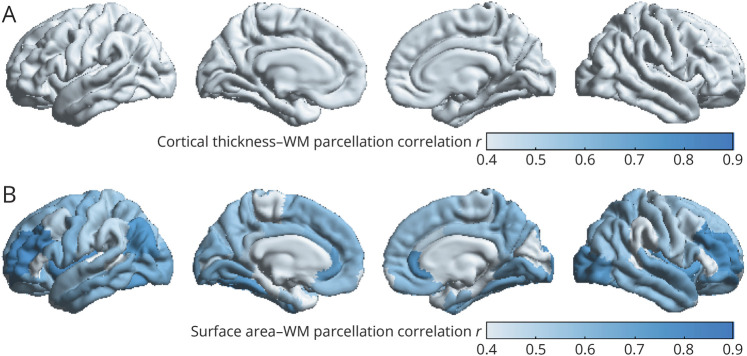
ROI-Based Correlations Between CT (A) and SA (B) and the Volumes of WM Segmented ROIs We observed significant correlations between the SA and the volume of WM ROIs at a whole-brain level. ROI = region of interest; SA = surface area; WM = white matter.

From the ROI-based correlation analysis between CT and SA values and WM ROIs volumes (eTable 3) in individuals with *STXBP1* encephalopathy, we observed modest correlations between the CT and the volume of WM ROIs (Figure 3A). The analysis between the SA and the volume of WM ROIs revealed strong correlations at a whole-brain level (Figure 3B), particularly in the frontal regions (e.g., left/right superior gyrus *r*_value_ = 0.900/0.898, *p*_value_ < 0.001/<0.001; left/right lateral orbitofrontal gyrus *r*_value_ = 0.917/0.914, *p*_value_ < 0.001/<0.001; left/right rostral middle frontal gyrus *r*_value_ = 0.939/0.894, *p*_value_ < 0.001/<0.001; left/right caudal middle frontal gyrus *r*_value_ = 0.908/0.843, *p*_value_ < 0.001/<0.001).

### Longitudinal Analysis on Single Individuals

Of the 4 patients for whom serial MRI scans available for comparative analysis (eFigure 4, eTable 4), 3 (patients 2, 8, and 10) were children with DEE whose MRIs were acquired 2–5 years apart between ages 4.4 and 12.5 years and one (patient 19) was an infant with EIDEE whose MRIs were acquired at ages 2.9 and 5.3 years. Longitudinal volumetric analysis of patients 2 and 10, acquired at comparable ages, revealed analogous rates of volume decline per year (−5.9% for patient 2, -4.4% for patient 10, eFigure 3A), but divergent WM growth rates (+14.0% in patient 2, +1.1% in patient 10, eFigure 3B), paralleling milder vs moderate ID.

### Comparisons Between Morphometric Analysis vs Gene Expression Patterns

We found *STXBP1* spatial expression levels (*ε*_value_), extracted by the Allen Human Brain Atlas, to be highly and homogeneously expressed throughout the cortex (eFigure 5A), with significant expression in the pericalcarine (left/right *ε*_value_ = 0.70/0.78), lingual (left/right *ε*_value_ = 0.68/0.78), lateral occipital (left/right *ε*_value_ = 0.66/0.78), cuneus (left/right *ε*_value_ = 0.70/0.87), and precentral (left/right *ε*_value_ = 0.63/0.59) gyri. Within the limbic circuit, amygdala (left/right *ε*_value_ = 0.645/0.330) and hippocampus (left/right *ε*_value_ = 0.689/0.664) exhibited the highest *STXBP1* expression (eFigure 5B).

After correlating the levels of *STXBP1* expression with morphometric cortical and subcortical alterations, we observed no statistically significant associations between gene expression levels and CT (*r*_value_ = 0.116, *p*_spin_ = 0.354, eFigure 5C) or SA (*r*_value_ = −0.037, *p*_spin_ = 0.769, eFigure 5D) alterations.

### Comparisons Between Morphometric Analysis vs Clinical and Genetic Variables in *STXBP1* Encephalopathy

By applying the multivariate statistical model to clinical variables of patients (eTables 5–10), we found more pronounced thickening in the parahippocampal gyri (eTable 5) related to earlier *age at seizure onset* (left/right *β*_2_ = −45.13/-30.94, *p*_value_ = 0.013/0.105). Associations with *PTV+del+splicing+start loss vs missense* variants (eTable 5) revealed that CT was higher in both hemispheres in patients with *missense* variants (left/right *β*_2_ = 2.80/2.81, *p*_value_ = 0.088/0.062), although not significantly. CT was significantly higher in the caudal middle frontal (left/right *β*_2_ = 3.17/3.44, *p*_value_ = 0.026/0.004), superior parietal (left/right *β*_2_ = 2.45/2.32, *p*_value_ = 0.040/0.036), precentral (left/right *β*_2_ = 2.91/5.09, *p*_value_ = 0.124/0.004), and postcentral (left/right *β*_2_ = 3.06/1.71, *p*_value_ = 0.037/0.153) gyri of patients with *missense* variants.

We found no significant associations between SA changes in patients and *age at seizure onset*, *intellectual disability*, or *PTV + del + splicing + start loss vs missense* variants (eTable 6). Neither did we observe significant associations between CT and SA changes in patients and *drug-resistant epilepsy*, *autism*, or use of *corticosteroid* at the time of MRI (eTables 7 and 8).

No significant associations emerged also between volumetric changes in subcortical structures and *age at seizure onset* (eTable 9). In patients with more severe *intellectual disability*, we observed a significant increase in volume of the lateral ventricles (left/right *p*_value_ = 0.049/0.030) and CSF (*p*_value_ = 0.019) and a no significant reduction in CC volume (central CC *p*_value_ = 0.056; middle anterior CC *p*_value_ = 0.072). There was more severe, although not significant, volumetric reduction at a whole-brain level in patients with *missense* compared with *PTV + del + splicing + start loss* variants (left/right WM *p*_value_ = 0.091/0.057, brain cortex *p*_value_ = 0.090, subcortical GM *p*_value_ = 0.086 volumes). We observed no significant associations between volumetric changes in subcortical structures and *drug-resistant epilepsy*, *autism*, or use of *corticosteroids* (eTable 10).

## Discussion

We compared quantitative neuroimaging measures of cortical and subcortical structures in patients with pathogenetic variants in the *STXBP1* gene with those of controls. Our findings indicate that *STXBP1* encephalopathy is associated with increased CT in the frontal and occipital lobes in both hemispheres. The SA was globally reduced, with more intense reductions in the frontal regions. Correspondingly, we observed a volumetric reduction of the hippocampal formation, WM and CC, and increased volume of the lateral ventricles and CSF. We noticed that the volumetric reduction of the WM was more pronounced in the regions underlying the frontal lobes and was regionally correlated with the reduction of SA. The WM exhibited areas of hypointensity, significantly more extended in patients than controls. Longitudinal analysis, only possible in 4 patients, highlighted that brain growth trends were lower than the average trend in the control cohort. In 2 children with similar age ranges (patients 2 and 10), we observed different patterns of WM growth because patient 2, with mild ID, exhibited a far higher rate of change (+14.0%) than patient 10 (+1.14%) who had moderate impairment.

Previously described MRI features in *STXBP1* encephalopathy only emerged from qualitative studies and included brain atrophy and abnormal CC and WM myelination.^12,13^ Six additional patients with either imaging suspected, or histopathologically proven, FCD have been reported.^14,15,31,32^ In both patients of our cohort with MRI findings suggestive of FCD, histopathology, performed after left anterior temporal lobectomy, confirmed dyslamination of cortical layers, consistent with FCD-I.

The WM volumetric and SA reductions we observed might represent the anatomic substrate for the impaired neurodevelopmental processes in *STXBP1* encephalopathy. The morphometric features of the cortical mantle (SA and CT) follow well-characterized trajectories during typical brain development.^33^ Cortical gyrification emerges primarily during fetal life, driven by the interaction between genetic programs, molecular signaling, and mechanical forces, and continues to refine during childhood and adolescence.^34^ Gyrification increases cortical SA within a constrained cranial volume and may improve connectivity by shortening the physical distance between cortical regions.^23^ Cortical SA is related to the number of cortical columns and reflects dynamic growth processes that emerge during gestation, by unfolding in a regionally and temporally heterogeneous manner.^35^ Cortical thickness is associated with the number of neurons within a cortical column and reflects key developmental processes, including neuronal migration, postmigrational organization, and cortical maturation.^22^ Cortical development follows a regionally specific and temporally ordered trajectory, with primary sensory and motor regions maturing first and higher-order frontal and temporal regions through prolonged windows of development.^35^ Longitudinal neuroimaging studies in typically developing populations consistently demonstrate progressive cortical thinning during late childhood and adolescence, particularly in association cortices, which may reflect experience-dependent synaptic pruning, increased intracortical myelination, and refinement of neural circuits underlying functional maturation.^36^ During the developmental period, synaptic pruning is a fundamental mechanism that supports the optimization and specialization of neural networks, facilitating more efficient information processing and the emergence of mature cognitive and behavioral functions.^37^ Deviations from normative CT and SA patterns have been associated with neuropsychiatric and neurodevelopmental conditions, which result in long-term functional impairments.

Monogenic encephalopathies may exhibit characteristic and reproducible patterns of altered brain morphology, reflecting gene-specific effects on neurodevelopmental programs. For example, in *PCDH19* encephalopathy, distinct patterns of cortical and WM alterations align with regional gene expression profiles and phenotypic aspects.^17^ Similarly, *SCN1A*-related disorders are associated with bilateral atrophic changes in temporo-limbic regions, with more pronounced involvement in Dravet syndrome compared with GEFS+, suggesting a combined influence of genetic etiology and seizure burden on brain development.^18^ The morphometric alterations we observed in *STXBP1* encephalopathy should be interpreted in relation to these established developmental principles. Compared with expectations derived from brain growth in controls, the identified deviations in cortical and WM measures suggest a disruption of regionally specific expansion and maturation processes, likely reflecting the interaction between the underlying genetic disorder, disease-related factors, and, likely, their duration.

The homogeneous expression of *STXBP1* in the cortex might explain the rather widespread alterations in SA and WM observed in our cohort, with no regional correlations between the levels of postnatal *STXBP1* expression and CT or SA modifications.

The anatomic asymmetry related to CT increases in the left hemisphere was unexpected in light of the homogeneous cortical expression of *STXBP1*.^25^ Several methodologic and neurobiologic factors might contribute to this finding. First, the statistical model includes handedness as a covariate. However, handedness information was unavailable for a substantial proportion of patients, while most of those with handedness information were right-handed. Given the known relationship between handedness and hemispheric specialization,^38^ this imbalance might have biased sensitivity toward detecting left-hemispheric effects. In addition, the relatively small sample size reduces statistical power, particularly for detecting more subtle or spatially distributed right-hemispheric changes. Age-related variations in CT are region-specific in healthy individuals, where hemispheric asymmetries in CT that may emerge at different stages of neurodevelopment and aging.^39^ Development of cortical asymmetry has been proposed to reflect differences in strength and lateralization of specialized functions between hemispheres.^40^ Alterations in cortical asymmetry in higher-order association cortices, such as the prefrontal cortex, have been observed in childhood-onset neurologic and neurodevelopmental disorders, including autism spectrum disorder.^41^ The marked rightward asymmetry observed in these regions may reflect atypical early brain growth trajectories characteristic of neurodevelopmental disorders. In our cohort with *STXBP1* encephalopathy, hemispheric asymmetries in CT might reflect the altered contribution of the STXBP1 protein to regional cortical development and WM organization, with functional consequences in the neurocognitive phenotype as well as in mechanisms underlying epileptogenesis.

The altered cortical patterns and WM reductions we observed in *STXBP1* encephalopathy might be associated with abnormal vesicle release at the synaptic level, which results in impaired dendritic maturation, formation of neuronal connections, and impulse propagation.^2^ Advanced development and maturation of neural connections, including dendritic arborization, is largely achieved in early childhood.^42^ Studies in human iPSC models have identified a pivotal role of *STXBP1* in the release of the membranous material necessary for dendritic growth.^43^ Impaired *STXBP1* function might reduce release of membranous material and thus compromise dendritic growth, neurite outgrowth/arborization, and maintenance processes. The development of functional neuronal connections also relies on proper synaptic function through neurotransmitter release,^44^ a process partially governed by neuronal SNARE complexes, where *STXBP1* plays a key role. These altered functional processes may underpin the decrease in WM volume, leading to constrain of cortical expansion (SA reduction) and secondary thickening of the cortical mantle, consistent with a mechanism of WM shrinkage, related to cell loss and damage to both axons and dendrites.^45^

ID is consistently observed across patients with *STXBP1* encephalopathy,^11,12^ and a more severe ID was associated with earlier onset of epilepsy in our cohort, consistent with findings from other studies.^46^ Considering that the estimated ICV did not significantly differ between patients and controls, ID in these patients might be partly explained by the more pronounced reductions in cortical SA and WM volume occurring in the frontal regions. Abnormal development of the frontal cortex may be particularly significant in *STXBP1* encephalopathy because it affects advanced executive functions such as problem-solving, decision-making, and social behavior.^47^ Frontal lobe dysfunctions are common across diverse neurodevelopmental disorders, regardless of etiology.^48^ Previous morphometric studies, including works from our group in *PCDH19* encephalopathy^17^ and *SCN1A*-related epilepsy,^18^ have demonstrated distinct patterns of structural alteration, such as predominant temporal rather than frontal involvement. Applying morphometric analyses to conditions with different etiologies but sharing clinical features, particularly other synaptopathies, may help determine whether the structural alterations we observed in *STXBP1* encephalopathy reflect a shared disease mechanism, or rather specific gene-driven features.

We observed comparable alterations in WM and SA in the occipital cortex, which could reflect more severe disruption of fiber bundles subserving the longest cortico-cortical connections, such as frontal-occipital pathways. At the core of these long-distance connections stands the inferior fronto-occipital fasciculus, connecting prefrontal to occipital cortices, whose function has been linked to motor cognition, attention, theory of mind, face perception, emotion recognition, and social behaviour.^49^ Thus, structural alterations involving such long-range WM tracts might further explain ID and possibly autistic traits in *STXBP1* encephalopathy.

Including our 2 patients, FCD-I features, whose hallmark is cortical dyslamination, have now been histopathologically demonstrated in 4 patients with *STXBP1* encephalopathy. If extended to the whole cortex, FCD-I changes would imply widespread abnormal lamination and connectivity.

Our study suggests that more severe ID might be associated with lower cortical and WM volumes. If confirmed in a larger series, this information might serve to exploit quantitative MRI studies for prognostic purposes, especially in early diagnosed patients.

Finally, we conducted genotype-phenotype analyses to establish whether the type of variant might per se impact morphometric patterns. We found that patients with *missense* variants exhibited more altered morphometric values at a whole-brain level when compared with those with *PTV + del + splicing + start loss* variants. We found significant cortical thickening and volumetric reduction of the CC and WM, which were more severe with *missense* variants. This finding might be related to the more severe effect expected for *missense* variants that could act through a dominant negative effect on the wild-type protein.^50^ Unlike *PTV* + *del* + *splicing* + *start loss* variants that result in a 50% reduction of functional protein (haploinsufficiency), certain missense variants result in the production of a mutant protein that can actively interfere with the wild-type syntaxin-binding protein. Previous research determined that these mutant proteins can act as defective molecular chaperones, leading to misfolding and subsequent aggregation of both the mutant and wild-type *STXBP1*, as well as other critical components of the SNARE complex.^45,50^ This “toxic gain of function” results in a more pronounced impairment of synaptic vesicle docking and neurotransmitter release than seen with simple haploinsufficiency and may in turn exacerbate the disruption of dendritic arborization and neurite maintenance—the structural processes we propose as the substrate for the observed increased CT and WM volume reduction in our cohort.

CC thinning is a common finding at brain MRI visual inspection in *STXBP1* encephalopathy^13^ and stands out in our cohort as well (7/24, 30%). Although such associations were only slightly significant, likely because of the small number of patients, the pattern of morphometric alterations might likely result from the combined neuroanatomic effect of the underlying etiology and seizure burden.

In conclusion, our morphometric analysis unveils some brain patterns, including widespread increase of CT, reduced frontal and occipital SA and WM, and reduced CC volumes, in *STXBP1* encephalopathy, which correlate with clinical and genetic features. Limitations in this study include retrospective analysis in a relatively small cohort with a wide age range. We considered age effects and evaluated age in the multivariate statistical models. MR images had not been obtained using a standardized protocol. To obviate these drawbacks, which are unavoidable when studying a rare condition, we implemented a validated pipeline for morphometric analyses that reduces inconsistencies arising from differences in MRI protocols across centers. Advanced diffusion MRI sequences for the modeling of the WM microstructure and neurite density, and tractography, may further elucidate the underlying impaired processes of neurite outgrowth and validate imaging biomarkers for prognosis and treatment stratification.

## Glossary

CC: corpus callosum
CT: cortical thickness
CV: cortical volume
DEE: developmental and epileptic encephalopathy
eICV: estimated intracranial volume
EIDEE: early infantile developmental and epileptic encephalopathy
FCD: focal cortical dysplasia
FLAIR: fluid attenuation inversion recovery
GM: gray matter
ID: intellectual disability
iPSC: induced pluripotent stem cell
PTV: protein-truncating variants
ROI: region of interest
SA: surface area
STXBP1: syntaxin-binding protein 1
